# Antifungal efficiency and cytocompatibility of polymethyl methacrylate modified with zinc dimethacrylate

**DOI:** 10.3389/fcimb.2023.1138588

**Published:** 2023-03-14

**Authors:** Jiali An, Yunpeng Song, Jing Zhao, Baohua Xu

**Affiliations:** Dental Medical Center, China-Japan Friendship Hospital, Beijing, China

**Keywords:** polymethyl methacrylate (PMMA), zinc dimethacrylate (ZDMA), surface characteristics, antifungal, cytocompatibility

## Abstract

**Objectives:**

Considering the high incidence rates of denture stomatitis, research that providing dental biomaterials with antifungal property are essential for clinical dentistry. The objectives of the present study were to investigate the effect of zinc dimethacrylate (ZDMA) modification on the antifungal and cytotoxic properties, as well as the variance in surface characteristics and other physicochemical properties of polymethyl methacrylate (PMMA) denture base resin.

**Methods:**

PMMA with various mass fraction of ZDMA (1 wt%, 2.5 wt% and 5 wt%) were prepared for experimental groups, and unmodified PMMA for the control. Fourier-transform infrared spectroscopy (FTIR) was applied for characterization. Thermogravimetric analysis, atomic force microscopy and water contact angle were performed to investigate the thermal stability and surface characteristics (n=5). Antifungal capacities and cytocompatibility were evaluated with Candida albicans (*C. albicans*) and human oral fibroblasts (HGFs), respectively. Colony-forming unit counting, crystal violet assay, live/dead biofilm staining and scanning electron microscopy observation were performed to assess antifungal effects, and the detection of intracellular reactive oxygen species production was applied to explore the possible antimicrobial mechanism. Finally, the cytotoxicity of ZDMA modified PMMA resin was evaluated by the 3-(4,5-dimethyl-thiazol-2-yl)-2,5-diphenyl-tetrazolium bromide (MTT) assay and live/dead double staining.

**Results:**

The FTIR analyses confirmed some variation in chemical bonding and physical blend of the composites. Incorporation of ZDMA significantly enhanced the thermal stability and hydrophilicity compared with unmodified PMMA (p < 0.05). The surface roughness increased with the addition of ZDMA while remained below the suggested threshold (≤ 0.2 µm). The antifungal activity significantly improved with ZDMA incorporation, and cytocompatibility assays indicated no obvious cytotoxicity on HGFs.

**Conclusions:**

In the present study, the ZDMA mass fraction up to 5 wt% in PMMA performed better thermal stability, and an increase in surface roughness and hydrophilicity without enhancing microbial adhesion. Moreover, the ZDMA modified PMMA showed effective antifungal activity without inducing any cellular side effects.

## 1 Introduction

As the population aged, improving the quality of life among them has caused great concern due to the difficulty aged people faced on a day-to-day basis. One of the most common issues in aged individuals is the teeth loss. Restorative dentistry has been the outstanding solution to restore such oral functions, and resin-based polymeric systems are widely applied in this area (Kostić et al., 2022). Poly methyl methacrylate (PMMA) is one of the most widely utilized of resin-based polymeric material due to its unique properties, including cost-effectiveness, well cytocompatibility, aesthetics, ease of manipulation, etc (Zafar, 2020). However, there are a number of concerns in the application of PMMA resin, such as its high porosity, surface hydrophobic and roughness (Matsuo et al., 2015; Walczak et al., 2020), which are susceptible to lead dental plaque deposition. In addition, poor denture hygiene contributes to inflammation in oral mucosa. All of these drawbacks are prone to lead denture stomatitis.

Denture stomatitis is a multifactorial oral disease, mainly caused by Candida albicans (*C. albicans*) infection. Candida-associated denture stomatitis has been found in up to 60% of denture wearers, being the most common oral mucosal issue related with denture base. To overcome such undesirable deficiency, considerable research about PMMA denture base resin modification have been studied, including incorporating nanoparticles, fibers or other fillers, utilizing copolymers to polymerize with PMMA resin matrix and curing in different conditions, etc (Gad et al., 2020; Karatepe and Ozdemir, 2020; Aati et al., 2022). The modified methods of PMMA based denture resin with monomer additives have been reported before (Kanie et al., 2004; Elzahar et al., 2022). For instance, the quaternary ammonium salts (QAS) monomer was a successful approach used in the restorative dentistry to overcome biofilm formation (Melo et al., 2014; Cao et al., 2018).

**Figure 1:**
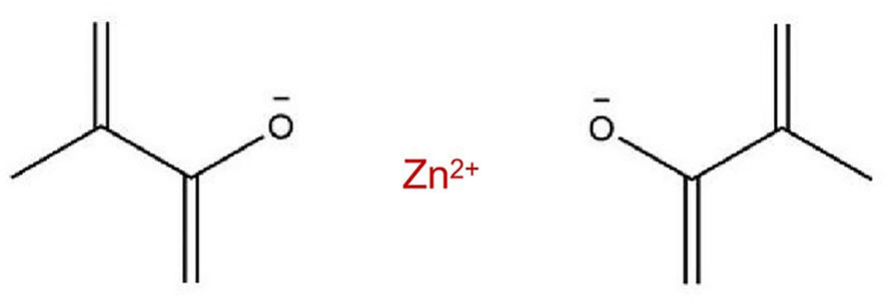
Molecular structure of zinc dimethacrylate.

In addition to this, a novel metal-containing methacrylate monomer has been applied in dental materials recently. The incorporation of metal methacrylate including silver, zinc, calcium and dibutyltin-containing methacrylate into dental resin matrix allowed the materials to exert novel bioactive properties (Rubin Cocco et al., 2018; da Silva Barboza et al., 2021). The ability of these monomers to become immobilized in the main polymer chain and antibacterial effects contribution by metallic elements make them possess potential application value in resin-based polymeric dental material modification. Previous studies have demonstrated that zinc-based dental materials performed broad-spectrum antibacterial activity and biological safety, and thus it is expected that zinc-containing methacrylate will show a feasible antimicrobial effect against oral pathogenic bacterial without impacting biocompatibility. Zinc dimethacrylate (ZDMA), existing in the form of homopolymerized polysalt particles with diameters around 20 ~ 100 nm (Ambrožič et al., 2011), is a kind of metal crosslinking monomer with unsaturated carboxylic groups (the molecular structure of ZDMA was displayed in **Figure 1**) (Chen et al., 2013a). Two methacrylate (MAA-) groups are connected to the divalent Zn^2+^ through ionic bonds, and under certain conditions (such as in the presence of oxidation reduction or peroxide), the C=C bonds in MAA- groups can polymerize both with the matrix and itself (Xu et al., 2016; Wu et al., 2022). The ZDMA monomer has showed remarkable performance in clinical dentistry as modification ingredients of composites (Henn et al., 2011; Cocco et al., 2020). For instance, dental resin adhesive containing ZDMA performed great capacities of matrix metalloproteinase 2 (MMP-2) inhibition without impacting resin bond strength (Henn et al., 2012). In our previous study, ZDMA was incorporated into PMMA denture base resin and it demonstrated that modified PMMA resin containing mass fraction of ZDMA less than 5 wt% showed enhanced mechanical properties and also displayed great antibacterial property against Streptococcus mutans (*S. mutans*). However, there is still a limited information about its antifungal performance, physicochemical characters, surface characteristics and cytocompatibility of such modification.

Therefore, the aim of this study was to further evaluate the effect of ZDMA at various mass fraction (1 wt%, 2.5 wt% and 5 wt%) on the thermal stability, surface roughness and hydrophilic/hydrophobic properties, antifungal and cytocompatibility performance of PMMA denture base resin. It was hypothesized that 1) incorporating ZDMA into PMMA resin would increase the surface roughness and hydrophilicity compared with unmodified PMMA; 2) incorporation of ZDMA will provide effective antifungal activity without inducing any cellular side effects.

## 2 Materials and methods

### 2.1 Preparation of specimens

The PMMA resin (Nissin, China) and ZDMA particles (Sigma-Aldrich, USA) used in this study were all commercially available. PMMA resin were prepared using the room temperature denture base polymerizing acrylic powder and liquid following the manufacturer’s instructions. To develop the new metal methacrylate modified PMMA resin, ZDMA was added at 1 wt%, 2.5 wt%, and 5 wt% to the PMMA monomer. Mass fraction greater than 5 wt% of ZDMA were eliminated considering that the preliminary study (An et al., 2022) has indicated a significant decrease in mechanical properties. Therefore, PMMA modified with ZDMA (1 wt%, 2.5 wt% and 5 wt%) were applied for the experimental groups, and unmodified PMMA for the control. In total, four groups were performed in this study. The manufacturing methods of the specimens for each group are described briefly below. Different mass fraction of ZDMA was added into acrylic powder and mixed with turbine mixer. After completely mix, acrylic liquid was added into the combined powder to form a paste which was then used to fabricate specimens for experimental groups. The acrylic powder and liquid were directly mixed to fabricate specimens for control group.

### 2.2 Characterization

#### 2.2.1 Fourier transform infrared spectroscopy (FTIR)

To investigate the effect of the ZDMA incorporation on the PMMA resin, the infrared spectra of transmittance of the specimens selected randomly from each group and ZDMA were recorded using FTIR (Nicolet iS5, Thermo Fisher Scientific, USA) with an attenuated total reflectance (ATR) sampling accessory. The wavenumber was set in the range of 550 to 4000 cm^−1^. For each spectrum 32 scans were recorded with resolution at 4 cm^-1^.

#### 2.2.2 Thermogravimetric analysis (TGA)

TGA (Q-50, TA instrument, USA) was applied for thermal stability characterization. The thermal stability of each sample was observed from ambient temperature to 500°C under nitrogen flow at a heating rate of 10°C/min. The weight loss experienced by the specimens as a function of temperature provided the kinetic data, thermal degradation behavior and rate of degradation.

#### 2.2.3 Surface roughness

Specimens (n=5 per group) with dimension of 2 mm in thickness and 10 mm in diameter were prepared for surface roughness examination. Atomic force microscopy (AFM, Multimode 8, Bruker Daltonics Inc, USA) was performed to detect the surface topography and roughness. AFM was used at a high resolution with a sharp silicon tip (0.5 N/m) in tapping mode. The surface topography of the specimens was obtained over an area 10 × 10 μm. The surface roughness of the specimens from each group was obtained with a systemic software (NanoScope Analysis 1.7, Bruker Daltonics Inc), and the data of arithmetic roughness (Ra) were compared. Ra represented the average distance from the roughness profile to the center plane of the profile.

#### 2.2.4 Water contact angle

The water contact angle of the specimens (n = 5) was measured using contact angle goniometer (OCAH200, Dataphysics, Germany) by quickly recording (less than 1 min) the images of ultrapure water droplets (3.5 μL) on the flat surface at 20°C. Contact angle measurements were repeated three times at different positions for each sample to obtain average values.

### 2.3 Antifungal activity

#### 2.3.1 Biofilm formation

For antifungal experiments, the specimens were fabricated as PMMA discs (diameter = 10 mm, thickness = 2mm) and prior to test, specimens were disinfected using 70% ethanol, washed in triplicate with phosphate buffered saline (PBS) and then placed under UV light for 30 mins. The antifungal activities of the specimens were evaluated by using *C. albicans* (ATCC 10231). *C. albicans* were provided by the China-Japan Friendship Hospital of Clinical Microbiology Laboratory, which were cultured in a Sabouraud Dextrose (SD) broth at 37 °C. Each PMMA disc was placed at the bottom of a 24-well plate and around 2 mL of *C. albicans* suspension (optical density, OD = 0.02, equivalent to 2 × 10^4^ CFU/mL) was added in each well and incubated for 24 h in a humid environment at 37°C under 95% air and 5% CO_2_ to form mature fungi biofilm on the surface of the samples.

#### 2.3.2 Colony-forming unit (CFU) counting

Following incubation, the specimens (n=5 per group) were washed twice with PBS to remove nonadherent fungi. The *C. albicans* biofilms adherent to the specimens were collected by scraping and sonication. The obtained biofilm suspensions were serially diluted, and 20 μL of diluted suspension was dropped onto SD solid medium plate and then incubated at 37°C for 24 h in anaerobic conditions. After 24 h, the colonies on the plate were counted for data analysis.

#### 2.3.3 Crystal violet (CV) assay for biofilm biomass

CV assay is the most commonly used quantitative technique for detecting biomass accumulation in microplate method. Specimens (n=5 per group) with *C. albicans* biofilms were rinsed with PBS to remove nonadherent fungi and then air dried for 20 min. The air-dried specimens were submerged in 100% methyl alcohol for 15 min for fixation and then stained with 0.1% CV solution (C8470, Solarbio, China) for 15 mins. The bound dye was extracted from the stained cells with 95% ethanol solution. Biomass accumulation was then quantified by measuring the optical density of the CV/ethanol extract at a wavelength of 600 nm in a microplate reader (SpectraMax M5, Molecular Devices, USA).

#### 2.3.4 Live/dead biofilm staining assay

The biofilm-coated specimens (n = 5 per group) were washed with sterile deionized water to remove non-adhered fungi. After rinsing with deionized water, the specimens with adhered *C. albicans* biofilms were stained with the LIVE/DEAD Yeast Viability kit (L7009, Thermo Fisher Scientific Inc, USA), followed by rinsing with deionized water to remove unnecessary fluorescence dye. The stained specimens were examined using an inverted epifluorescence microscope (Eclipse TE2000-S, Nikon, USA).

#### 2.3.5 Scanning electron microscopy (SEM)

The biofilm-coated specimens (n = 5 per group) were employed to observe *C. albicans* adhesion population and biofilm morphology by SEM (Phenom ProX, Thermo Fisher Scientific Inc, USA). Each specimen was fixed with 2.5% glutaraldehyde at 4°C overnight, and then rinsed with PBS. After fixation, the samples were dehydrated in a graded series of ethanol solutions (30%, 50%, 70%, 80%, 90% and 100%), desiccation, sputter coated with Au−Pd alloy and then inspected by SEM.

#### 2.3.6 Detection of intracellular reactive oxygen species (ROS) production

Intracellular ROS levels in *C. albicans* adhering to the specimen surface were measured by a fluorometric kit (Solarbio, China) using 2,7-dichlorofluorescein diacetate (DCFH-DA) probe as a ROS indicator as previously described (Pourhajibagher et al., 2020). Briefly, the *C. albicans* biofilms attached to the surface of specimens (n = 10 per group) were collected, washed with PBS for three times and then resuspended in PBS by adjusting the cell density to 10^6^ CFU/mL. DCFH-DA at concentration of 10 μmol/L was added to the fungus suspension and incubated for 20 mins at 37 °C. The microbial cells were then collected by centrifugation at 12000 g for 5 mins and washed with PBS to remove any unnecessary DCFH-DA. The fluorescence spectrophotometer (Thermo Fisher Scientific Inc, USA) at an emission wavelength of 535 nm and an excitation wavelength of 488 nm was used to determine the fluorescence intensity of the target cells and the inverted epifluorescence microscope was applied for fluorescence observation.

### 2.4 Cytotoxicity

#### 2.4.1 Cell culture

Human oral fibroblasts (HGFs; PCS-201–030, ATCC, USA) were cultured in fibroblast basal medium (FBM; SC 2301, WHELAB, USA) supplemented with 2% fetal bovine serum, 1% penicillin/streptomycin and 1% fibroblast growth supplement. The culture was incubated with 5% CO_2_ at 37°C under saturated humidity until 95% confluence was achieved. Cells between the 4 and 6 passages were used for subsequent experimental procedures.

#### 2.4.2 Preparation of the extract

In total, 10 specimens were prepared for each group, leading to a total surface area of 13.2 cm^2^. The ratio of the specimen surface to the medium volume was 1.25 cm^2^/mL. The specimens were sterilized and then eluted with fresh FBM at 37°C for 24, 48 and 72 h. The obtained extracts were passed through 0.22 μm filters for the next cytotoxicity tests.

#### 2.4.3 Detection of cell viability

To evaluate the cell viability of the new metal monomer modified PMMA, a 3-(4,5-dimethyl-thiazol-2- yl) -2,5-diphenyl-tetrazolium bromide (MTT) kit (M1020, Solarbio, China) was used according to ISO 10993. The cells were collected and seeded at a density of 10^5^ cells/mL in 24-well plates. After proliferation, 100 μL aliquots of the various extracts obtained from each group were added to 96-well plates. After 24 h of cell culturing, the medium was removed and MTT solution was added. The incubation was cultured at 37°C for 4 h, and the OD value of the formazan product at 450 nm was measured using a microplate reader, since the absorbance of formazan reflects the cell metabolism. Cells without the extracts were cultured as negative control and the extracts containing medium and MTT solution without cells were cultured as blank group. The cell viability value was calculated using the following equation:


$$\text{Cell Viability }=\;\left[\text{O}{\text{D}}_{\left(\text{t}\right)}–\text{ O}{\text{D}}_{\left(\text{blank}\right)}\right]/\left[\text{O}{\text{D}}_{\left(\text{nc}\right)}–\text{ O}{\text{D}}_{\left(\text{blank}\right)}\right]\;\times \;100\%$$

where OD_(t)_ is the OD value of the extracts from all groups, OD_(blank)_ is the OD value of the blank group, and OD_(nc)_ is the OD value of the negative control group.

#### 2.4.4 Live/dead double staining

HGFs were seeded at a density of 10^5^ cells/well in 24-well plate. The extract from each group was added after 4 h of proliferation. After 24 h of incubation, a live/dead staining kit (L10119, Thermo Fisher Scientific Inc, USA) was utilized to assess the cytotoxicity of the extracts. Calcein-AM was a staining reagent for fluorescently labeling living cells with green fluorescence, and its working concentration was 1 μM. In addition, PI (3 μM) only stained dead cells and excited red fluorescence. Finally, dyed cells were visualized through inverted epifluorescence microscope.

### 2.5 Statistical analysis

The statistical data are expressed as the mean ± standard deviation. Statistical analysis was performed by using a statistical software program (IBM SPSS Statistics, v22.0 for Windows, IBM Corp, USA). Dependent variables were evaluated by one-way analysis of variance (ANOVA) followed by the Tukey honestly significant difference *post hoc* test (α=.05) for this study, given that the data were consistent with a normal distribution and variance homogeneity.

## 3 Results

### 3.1 Characterization

#### 3.1.1 FTIR

**Figure 2:**
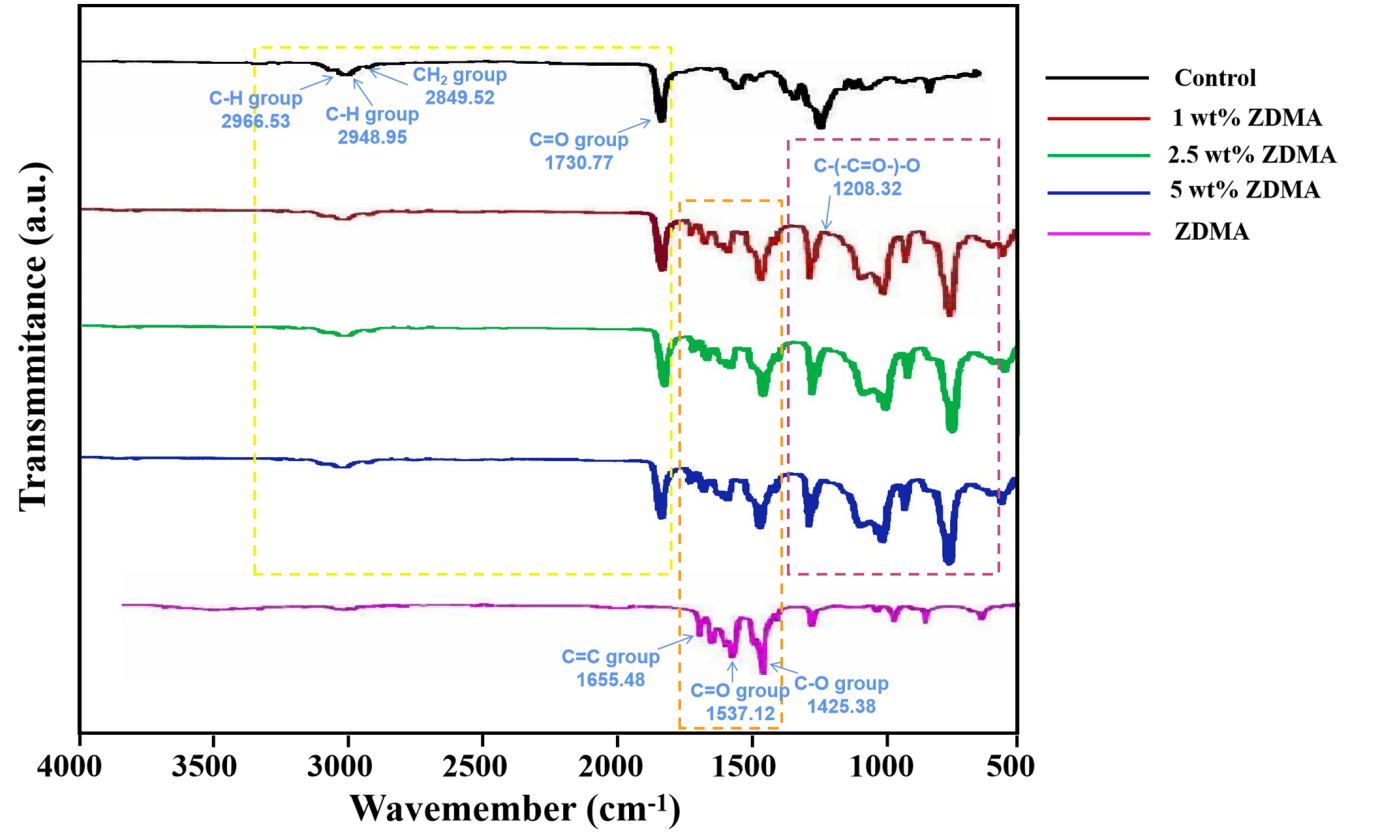
FTIR spectra of unmodified PMMA, ZDMA modified PMMA and ZDMA.

**Figure 2** shows the FTIR spectra (550 - 4000 cm^−1^) of the unmodified PMMA, ZDMA modified PMMA and ZDMA particles. The spectra of all groups were similar which exhibited the characteristic absorption peaks of PMMA (Karatepe and Ozdemir, 2020; Akhtar et al., 2021), such as the IR peaks at 2996.53 cm^-1^ and 2948.95 cm^-1^ representing the C-H asymmetric and symmetric stretching absorption peaks, that at 2849.52 cm^-1^ and 1730.77 cm^-1^ representing the CH_2_ and C=O stretching vibration absorption peak respectively. After ZDMA incorporation, ZDMA modified PMMA exhibited the characteristic absorption peaks of ZDMA, including the IR peaks at 1655.48 cm^-1^, 1610.36 cm^-1^ and 1425.38 cm^-1^, etc. The infrared spectra about ZDMA can be described that, the C=O and C-O have been homogenized due to the existence of Zn^2+^ which leading the two chemical bonds move to the absorption peaks of 1537.12 cm^-1^ and 1425.38 cm^-1^, and C=C bonds move its position to 1655.48 cm^-1^ due to conjugated with C=O (Ambrožič et al., 2011; Chen et al., 2013a). In addition, certain effects were noticed after ZDMA modification, such as transmittance being modified due to the cross-linking and physical blend leading the formation of new chemical bonds. One of the noteworthy IR peaks among the transmittance was the stretching vibration peak at 1208.32 cm^-1^. The generation of IR peak at 1208.32 cm^-1^ was due to the open of C=C double bond in ZDMA which confirmed the cross linking of ZDMA in the composite (Ambrožič et al., 2011; Chen et al., 2013b).

#### 3.1.2 TGA

**Figure 3:**
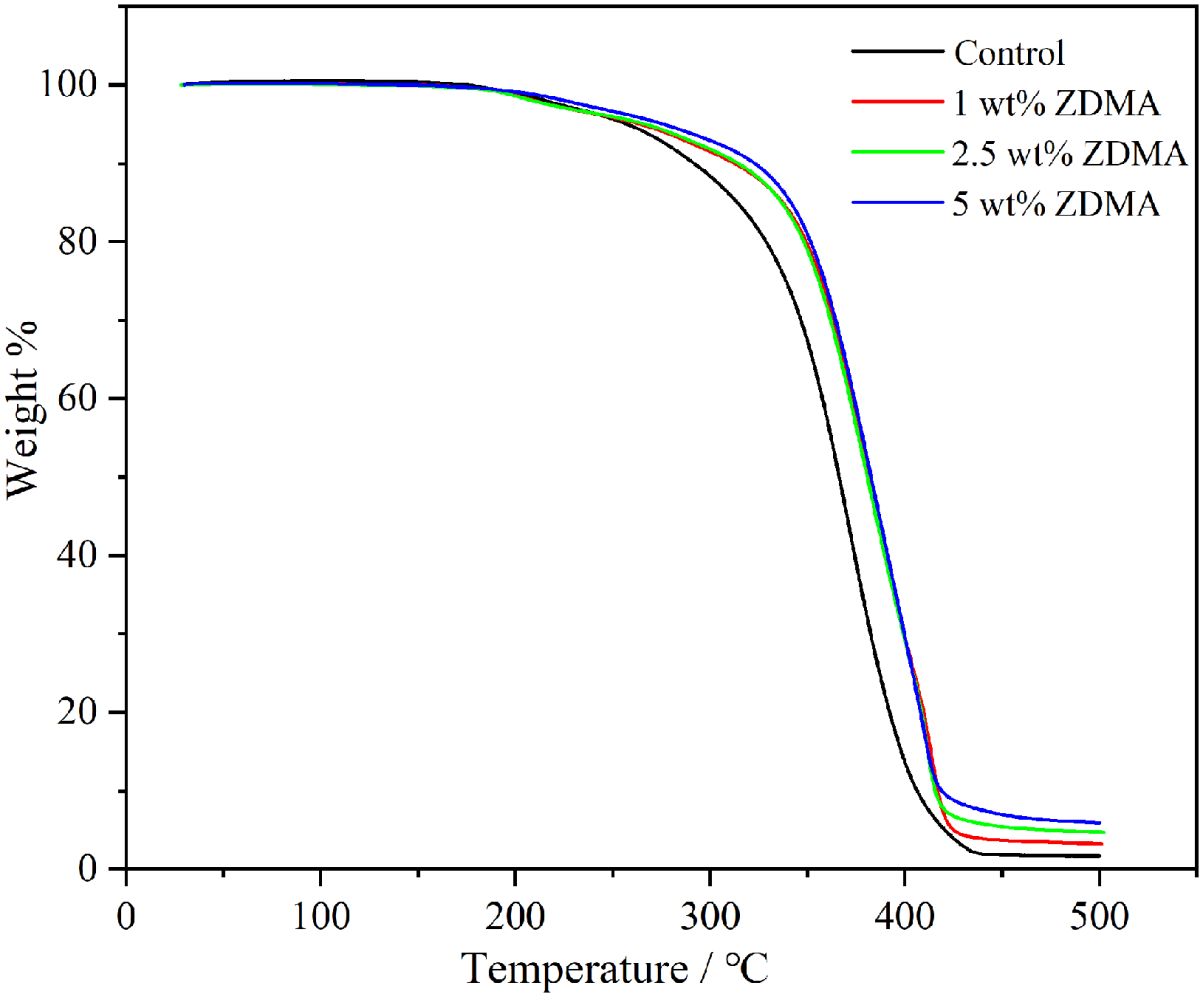
TGA weight loss curves of each group.

In order to evaluate the thermal stability of each sample, TGA analysis has been carried out up to 500°C at the heating rate of 10°C/min in nitrogen atmosphere. The weight loss curves are shown in **Figure 3** and the comparative study of TGA data for each group are displayed in **Table 1**. The thermogram of all samples displayed a three-step thermal degradation behavior (Adnan et al., 2021). The first weak weight loss step corresponds to chain scission resulting from hydrogen bonds. The second step is attributed to the scissions of the chains at the unsaturated ends while the later beginning of weight loss is due to the polymeric chain random scission. It is noticeable that the decomposing stage of ZDMA modified PMMA were significantly postponed compared with the unmodified PMMA. As shown in **Table 1**, the degradation temperature range in the three-step weight loss of ZDMA modified PMMA were all higher than that of the unmodified PMMA, and with the ZDMA mass fraction increased, the degradation temperature range increased gradually. T_5_, T_10_ and T_50_ representative the temperature of mass loss fraction at 5 wt%, 10 wt% and 50 wt%, respectively. In order to evaluate thermal stability, a reference point was selected as criterion, usually, at 5 wt% mass loss according to literature (Vedhanayagam et al., 2020). The TGA results indicated that T_5_ of ZDMA modified PMMA were all higher than unmodified PMMA and increased with the ZDMA mass fraction, implying that the thermal stability of ZDMA modified PMMA had been improved.

**Table 1:** Comparative study of TGA data for each group.

Groups	Stage	Temperature Range (°C)	Weight Loss	T_5_ (°C)	T_10_ (°C)	T_50_ (°C)
Control	I	195.81 ~ 292.35	11 wt%	257.04	291.67	366.28
	II	292.35 ~ 386.84	75 wt%			
	III	386.84 ~ 438.23	97 wt%			
1 wt% ZDMA	I	194.67 ~ 292.04	9 wt%	264.05	298.09	380.14
	II	319.37 ~ 388.29	59 wt%			
	III	388.29 ~ 460.26	96 wt%			
2.5 wt% ZDMA	I	192.51 ~ 323.31	12 wt%	266.67	314.37	380.17
	II	323.31 ~ 389.55	60 wt%			
	III	389.55 ~ 466.16	96 wt%			
5 wt% ZDMA	I	202.29 ~ 324.74	10 wt%	275.96	324.74	381.29
	II	324.74 ~ 387.69	56 wt%			
	III	387.69 ~ 483.82	93 wt%			

#### 3.1.3 Surface roughness

**Figure 4:**
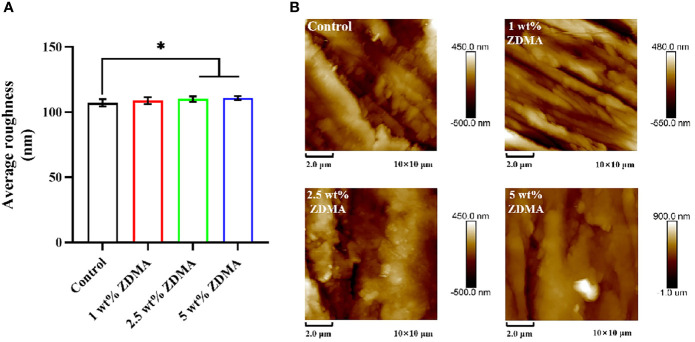
Surface roughness of each group. **(A)** Means ± standard deviations for Ra values of each group (*p < 0.05). **(B)** Representative AFM image of each group.

The surface roughness was examined *via* AFM, and **Figure 4** illustrates representative images and Ra values of each group. The control group showed the lowest Ra value (107.2 ± 2.76 nm). Compared with the control group, ZDMA modified PMMA groups have shown higher Ra values, 108.8 ± 2.59 nm, 110.1 ± 2.24 nm and 110.8 ± 1.63 nm, and there was an absence of statistically significant difference among these three experimental groups (p > 0.05). The 5 wt% ZDMA group (110.8 ± 1.63 nm) displayed the highest Ra values and significantly higher than the control group (p < 0.05).

#### 3.1.4 Water contact angle

**Figure 5:**
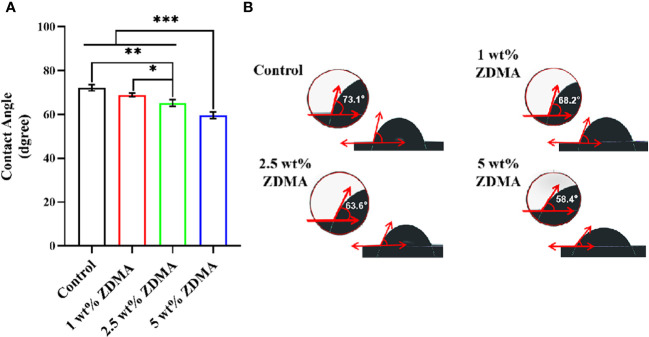
The water contact angle measurements of different specimens. **(A)** Means ± standard deviations for contact angle of each group (*p < 0.05, **p < 0.01, ***p < 0.001). **(B)** Representative contact angle image of each group.

The water contact angles of each group are exhibited in **Figure 5**. The control group showed the highest hydrophobicity with a contact angle of around 72.1 ± 1.4°. After ZDMA was incorporated into PMMA, as the mass fraction increased (1 wt%, 2.5 wt% and 5 wt%), the contact angle decreased gradually. The contact angle of 5 wt% ZDMA group (59.57 ± 1.53°) was the lowest among these groups and significantly lower than other groups (p < 0.001).

### 3.2 Antifungal activity

#### 3.2.1 CFU

**Figure 6:**
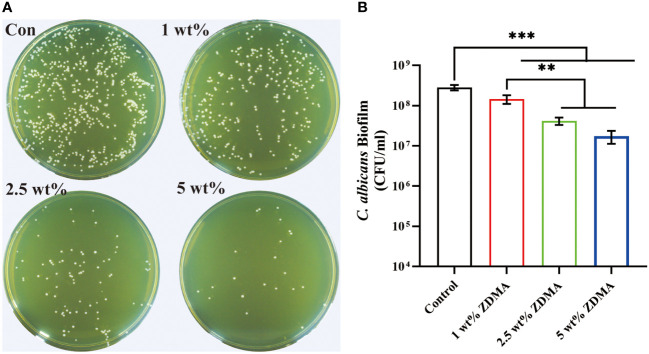
The number of *C. albicans* colonies detached from each group. **(A)** Representative images of *C. albicans* colonies of each group. **(B)** Means ± standard deviations for *C. albicans* colonies of each group ( **p < 0.01, ***p < 0.001).

The amount of *C. albicans* colonies (CFU/ml) from each group are shown in **Figure 6**. The *C. albicans* colonies of the control group were much denser than those of other experimental groups (p < 0.001). For the specimens with ZDMA added, those with 5 wt% ZDMA mass fraction PMMA showed the lowest number of colonies among all groups, about 2-fold lower than the control.

#### 3.2.2 Crystal violet assay

**Figure 7:**
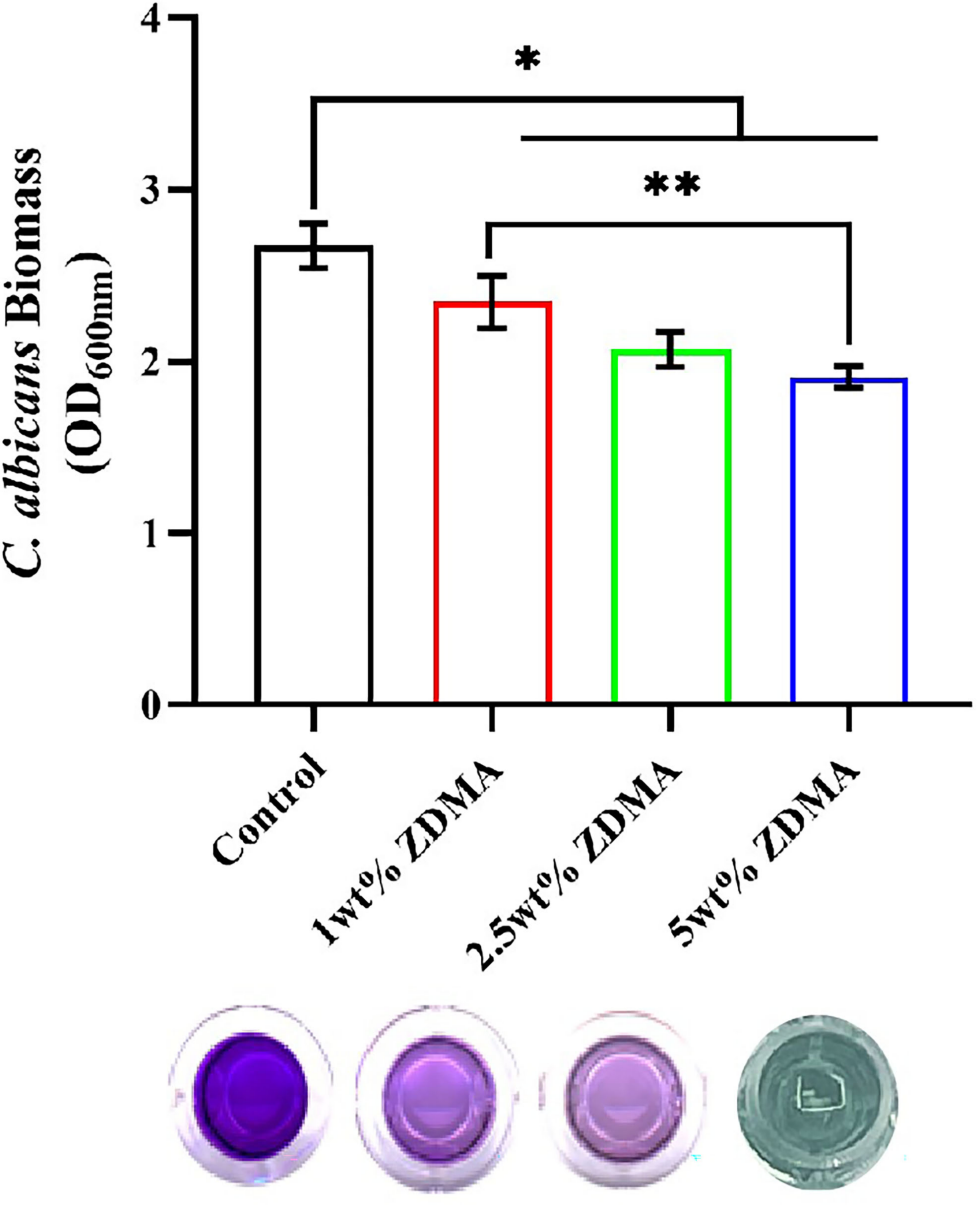
The visualization of *C. albicans* biofilm formation and means ± standard deviations for *C. albicans* biomass accumulation of the specimens for each group (*p < 0.05, **p < 0.01).

The *C. albicans* biomass accumulation was indicated by CV assay and the results are shown in **Figure 7**. The variance of the *C. albicans* biomass accumulation for each group was similar to the CFU results. Compared with the control group (2.67 ± 0.01), the biomass accumulation reduced with the ZDMA incorporation. When the mass fraction of ZDMA up to 5 wt%, the biomass accumulation was the lowest (2.07 ± 0.06), reduced significantly compared with the control group (p < 0.05).

#### 3.2.3 Live/dead staining

**Figure 8:**
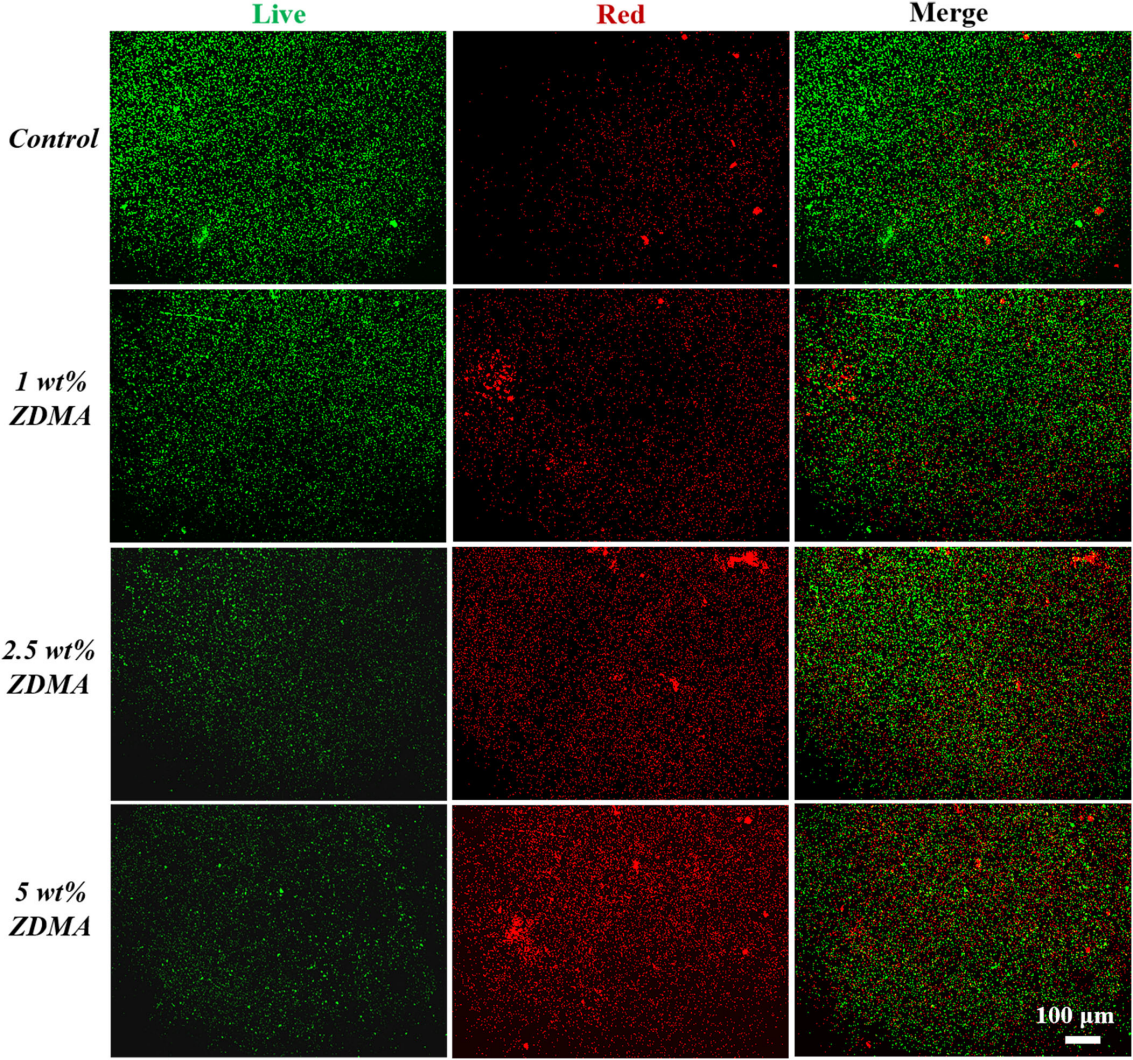
Representative live/dead staining images of *C. albicans* biofilms on specimens of each group.

Representative live/dead staining images of the *C. albicans* biofilm on the surface from each group are shown in **Figure 8**. The live microbial cells were stained green while the microbial cells with compromised membranes were stained red, and the overlap of live and compromised membranes were stained orange. The control group was primarily covered with live microbial cells with green staining, while the proportion of red staining significantly increased in ZDMA modified PMMA groups, indicating that dead microbial cells obviously increased.

#### 3.2.4 SEM observation

**Figure 9:**
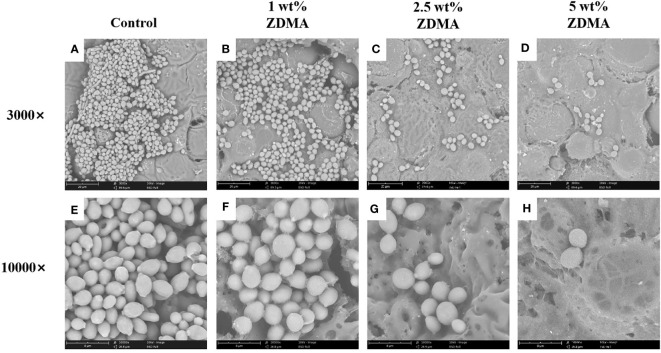
Representative scanning electron microscopy images showing morphologic changes of *C. albicans* on the specimens of each group: Images **(A–D)** represent SEM observation at 3000 magnifications. Images **(E–H)** represent SEM observation at 10000 magnifications.

The morphology and distribution of the *C. albicans* adhered to the surface of each group observed by SEM are shown in **Figure 9** and it is obvious that the biofilm of the control group was much denser than those in experimental groups. The *C. albicans* in the control group shrank less, exhibited a more complete plump shape and formed more contiguous and thicker biofilm covering the entire acrylic surface. The amounts of adhered *C. albicans* on the ZDMA modified PMMA surface reduced significantly and decreased as the mass fraction of ZDMA increased. Compared with the control group, ZDMA modified PMMA displayed a sparse and less *C. albicans* compact biofilm distributed on its surface.

#### 3.2.5 Detection of intracellular ROS production

**Figure 10:**
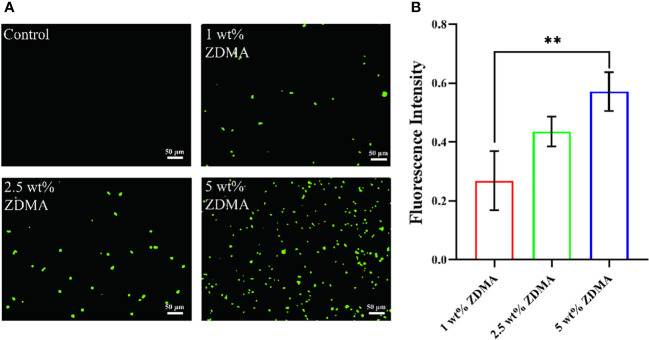
The expression of intracellular ROS in *C. albicans*. **(A)** Representative ROS fluorescence staining images from each group. **(B)** Means ± standard deviations for fluorescence intensity of each group (**p < 0.01).

The levels of intracellular ROS in *C. albicans* were detected by DCFH-DA fluorescent probe in the present study. DCFH-DA itself has no fluorescence and it can pass through the cell membrane freely. Once inside, DCFH-DA is first hydrolyzed into DCFH by lipase, and then be oxidized to DCF, with strong fluorescence, by ROS. Accordingly, the production of ROS in cells can be examined by the DCF fluorescence intensity. The fluorescence intensity and representative staining images of each group are displayed in **Figure 10**. As shown in **Figure 10A**, there was almost no fluorescence production when the co-culture of *C. albicans* with unmodified PMMA. In the experimental groups, the fluorescence amount and intensity significantly increased with the incorporation of ZDMA. The data of fluorescence intensity detecting by fluorescence spectrophotometer are shown in **Figure 10B**. No fluorescence expression detected in the control group. The ROS fluorescence intensity in *C. albicans* adhering to the specimen surface of experimental groups was in direct proportionate to the increased mass fraction of ZDMA. The fluorescence intensity of 5 wt% ZDMA group was the highest, significantly higher than 1 wt% ZDMA group (p < 0.01).

### 3.3 Cytotoxicity

**Figure 11:**
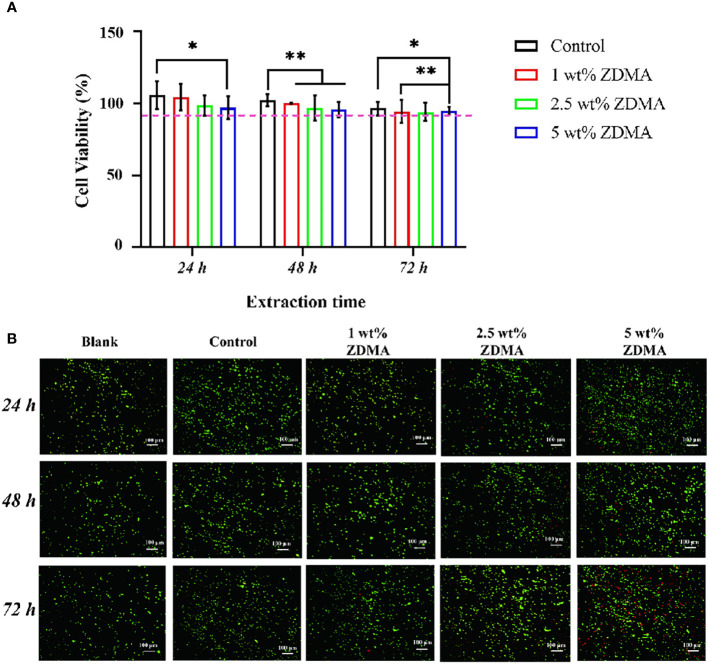
Cytotoxicity results of the extracts from each group. **(A)** The cytotoxicity of the extracts from each group determined by MTT assay (*p < 0.05, **p < 0.01). **(B)** Representative images of live/dead double staining of HGFs seeded with the extracts for each group and different extracting time.

The *in vitro* cell viability and cytotoxic effect of the extracts from each group against HGFs were measured and imaged as shown in **Figure 11**. The relative cell viability reduced with the mass fraction of ZDMA increased among the experimental groups compared with the control group. The 5 wt% ZDMA group exhibited the lowest cytocompatibility, decreased by 19.1% at most and significantly lower than the control group (p < 0.05). With the extension of extracting time, the relative cell viability of each group all decreased. In the present study, the relative cell viabilities were all greater than 90%. The effect of the extracts from each group in different experimental time on HGFs cell proliferation activity were determined by live/dead cell staining kit, and live cells were dyed green with calcein AM while dead cells displayed red due to propidium iodide. Live/Dead double staining assay (**Figure 11B**) presented that the live cells (green) accounted for more than 90% of the total cells in all groups, but it was obvious that with the increase of mass fraction of ZDMA, the proportion of dead cells (red) increased significantly, particular in 5 wt% ZDMA group.

## 4 Discussion

PMMA is the most commonly utilized polymers in clinical dentistry due to its great physiochemical properties and cytocompatibility (Zafar, 2020). However, the use of PMMA resin in the oral provides more surfaces for microorganism adhesion and alters biofilms distribution, impacting its application and oral health (Gad et al., 2017). Many attempts have been proposed to overcome such deficiency, such as incorporating QAS monomer into PMMA resin matrix. Such quaternary ammonium compound incorporation was reported to be cytotoxic and impacting mechanical properties (Beyth et al., 2008; Pei et al., 2018). To address these issues, in our previous study, ZDMA, a versatile crosslinker, was incorporated into PMMA matrix to generate a novel kind of metal methacrylate modified PMMA resin, and it was found that when the mass fraction of ZDMA was no more than 5 wt%, ZDMA provided modified PMMA antibacterial action against *S. mutans* and mechanical properties enhancement. In this study, surface characteristics, antifungal property, cytocompatibility and other physiochemical properties were further detected, and the results indicated that the incorporation of ZDMA significantly affected *C. albicans* adhesion and activity, without impacting its cytocompatibility.

FTIR results in **Figure 2** displayed that all groups contained the characteristic absorption peaks of PMMA, demonstrated that the main structural component of all specimens is the PMMA polymer (Muhammad et al., 2022). The appearance of new absorption peaks in experimental groups at 1425.38 cm^-1^, 1537.12 cm^-1^ and 1655.48 cm^-1^ related to ZDMA (Chen et al., 2013a) also confirmed itself blend modification with PMMA matrix. The resulting modified PMMA resin not only have both PMMA and ZDMA characteristic chemical bonds information, but also showing some new absorption peaks in the range of 1400 cm^-1^ to 550 cm^-1^. The appearance of these new transmittance revealed some chemical bonding and physical blend between ZDMA and PMMA in those modified composites (Elmergawy et al., 2021). One of the noteworthy IR peaks among the transmittance was the stretching vibration peak at 1208.32 cm^-1^. The C=C double bond of ZDMA opened and then the single bond C-(-C=O-)-O generated (Ambrožič et al., 2011; Chen et al., 2013b). The stretching vibration peak around 1208 cm^-1^ represented the generation of the single bond C-(-C=O-)-O. In the present FTIR spectrum (**Figure 2**), the appearance of IR peak at 1208.32 cm^-1^ revealed cross linking of ZDMA monomer in the composite. However, the FITR spectrum only confirmed the generation of cross linking in ZDMA, more specific results about cross linking degree or efficiency should be justified by other characteristic tests.

The thermal characteristics enhancement of the ZDMA modified PMMA resin could be observed studying the TGA results. ZDMA, a kind of ionic crystalline solid, is high in melting point and stable in chemical structure due to the strong interaction between positive and negative charges of the two ionic crosslink bonds (Xia et al., 2021). As it turns out that the second and third stage of the thermal degradation in all ZDMA modified specimens postponed, and the delay degradation stage may result from the incorporation of ZDMA which could increase the bond energy and stability of the composites (Chrysafi et al., 2020). Water contact angle is often used as an indicator for hydrophilic/hydrophobic properties of polymers surfaces. As illustrated in **Figure 5**, the contact angle of ZDMA modified PMMA were significantly lower compared with the unmodified PMMA, implying the incorporation of ZDMA increase the surface hydrophilicity of the composites. This may be partly attributed to the molecular structure of ZDMA. The hydrophilic functional groups, including carboxyl, hydroxy, carboxylate and acylamino groups, etc, impart hydrophilicity to the polymers (Zimudzi et al., 2018). ZDMA is a kind of unsaturated metal carboxylates, containing two unsaturated carboxylate groups (Chen et al., 2013b; Xia et al., 2021) (its structure was shown in **Figure 1**). The incorporation of ZDMA provided more hydrophilic groups than unmodified PMMA which may result in the hydrophilicity increase (Nonkumwong et al., 2018).

The application of PMMA denture base resin placing into oral changes the oral environment as well promotes the microbials formation and attachment on the composites surface. The most common isolated oral microorganisms from PMMA denture base resin are *C. albicans*, which is primarily leading to denture stomatitis, and *S. mutans*, which is mainly responsible for dental caries (Lapinska et al., 2020). Some studies have reported that *S. mutans* have a close association with increasing *C. albicans* biofilm formation (Pereira-Cenci et al., 2008). Therefore, it is crucial for ZDMA modified PMMA denture base resin to detect the potential antibacterial and antifungal effect against *S. mutans* and *C. albicans*. In our previous study, ZDMA modified PMMA has been proved to process antibacterial property against *S. mutans* while this study evaluated the antifungal effect on *C. albicans*. The amount of adherent and the activity of *C. albicans* on the ZDMA modified PMMA surface was significantly reduced by the addition of ZDMA, especially with the ZDMA mass fraction increased (as shown in **Figures 5** **–** **8**). The antifungal activity of ZDMA modified PMMA resin against *C. albicans* could be generally considered to be the contribution of the divalent Zn^2+^ connected with two methacrylate groups in ZDMA molecular structure. Zinc-containing dental materials, such as zinc oxide-eugenol cements and zinc ion coating implants, have been reported to show great antimicrobial performance against microbial strains (Arun et al., 2020; Yin et al., 2021). The ROS formation activated by Zn^2+^ is the key in microorganism inhibition (Wu et al., 2009). The excited electron catalyzed by Zn^2+^ react with the oxygen absorbed on the Zn^2+^, and then reduce the oxygen into ROS or hydroxyl radicals. Such ROS or hydroxyl radicals with strong redox activity would lead to the cell membrane and protease structure damage, microbial cell dysfunction, and ultimately exerting antimicrobial effects (Vedhanayagam et al., 2020; Yang et al., 2021). In the present study, the intracellular ROS fluorescence intensity were detected in *C. albicans* adhering to ZDMA modified PMMA surface but no fluorescence intensity in *C. albicans* on unmodified PMMA surface. These results uncovered that the ZDMA modified PMMA exerts its antifungal activity through Zn^2+^ induced ROS accumulation in *C. albicans*.

Moreover, apart from the active antimicrobial ingredients, another primary influence upon microorganism adhesion were the physicochemical property of the dental material surface, including van der Waals force, surface energy, hydrophobicity and roughness, etc (Lee et al., 2018; Kallem et al., 2021). Surface roughness is one of the key determinant factors in dental materials application. There are many previous studies have reported that surface roughness could substantially affect bacterial adhesion on the surface of polymers, susceptible to cause oral health issues (Zou et al., 2017). It was determined that the Ra value over 0.2 µm leading to an increase of microorganism attachment (Al-Harbi et al., 2019). In the present study, the surface of each sample remained unpolished to make the comparison even handed between groups. The AFM results in **Figure 4** showed that the Ra values of ZDMA modified PMMA increased with the mass fraction of ZDMA increase. Among these experimental groups, the Ra values of 5 wt% ZDMA group increased by 3.4% compared with unmodified PMMA. Although, ZDMA incorporation increased the surface roughness of modified PMMA resin, it remained significantly below the suggested threshold of dental materials (Ra ≤ 0.2 µm) to avoid negative impact in application, such as microorganism adhesion and accumulation. In addition, an associated change with surface characteristic was the hydrophilicity enhancement in ZDMA modified PMMA resin. The hydrophilicity/hydrophobicity significantly influence the microorganism adherence and accumulation on materials surface (Hamid et al., 2021). The hydrophobic interaction occurs between the microbial surface and PMMA resin, leading the microbial cells to overcome the initial electrostatic repulsive forces between them (Krasowska and Sigler, 2014; Aati et al., 2022), thereby enhancing microbial biofilm adherence over the substrates. Another plausible explanation could be that hydrophobic surfaces favors proteins accumulation which provide specific binding sites for microbiomes and thus accelerating and facilitating their adhesion as well (An and Friedman, 1998). The surface hydrophilicity enhancement would lead to the formation of a tight water layer that create a physical barrier and then inhibit microbial adhesion. The results in the present study were consistent with the aforementioned studies, the amount of *C. albicans* adherence to the surface of the modified PMMA reduced significantly with the surface hydrophilicity enhancement, that confirmed by the CFU counting, biomass accumulation examining by CV assay, and the evident biofilm observation through SEM. The incorporation of ZDMA into PMMA matrix increased the composites hydrophilicity and then reduced the *C. albicans* attachment on its surface.

Biocompatibility of dental materials is a critical consideration for application in clinical (Caldas et al., 2019). In the present study, the inhibition about HGFs cell viability with ZDMA modified PMMA enhanced with the ZDMA mass fraction increase and the extension of extracting time. This may partly result from the incomplete cured residual monomer (Ausiello et al., 2013), including MMA and ZDMA, leaching out from resin matrix. The unpolymerized monomer can induce adverse effects in biological tissues through cell DNA damage, inhibiting cell cycle, et al (Issa et al., 2004). Apart from this, ZDMA modified PMMA resin can not only induce *C. albicans* to generate ROS to exerts its antifungal activity but also induce normal cells to product ROS leading to itself apoptosis, and this may impact biocompatibility. The results of cytotoxicity in the present study supports our inference about the inhibition in cell viability. In general, despite an inhibition in cell viability compared the unmodified PMMA resin, it was still all above 90% in all ZDMA modified PMMA, considered well biocompatibility according to the International Organization for Standardization (ISO) 10993-5 standard (Walters et al., 2016).

The achieved results in the present study verified the hypotheses that incorporating ZDMA into PMMA resin increased the hydrophilicity and roughness without enhancing microbial adhesion compared with unmodified PMMA; incorporating ZDMA into PMMA resin achieved great antifungal effects and without inducing any cellular side effects.

## 5 Conclusion

A novel metal methacrylate monomer modified PMMA denture base resin was developed in the present study. With the ZDMA mass fraction increased (up to 5 wt%), the thermal stability and surface hydrophilicity enhanced significantly, and the surface roughness also increased while remained below the recommended threshold. Moreover, ZDMA modified PMMA resin showed great antifungal activities without inducing any cytotoxic effects. Therefore, the modification of PMMA denture base resin with ZDMA monomer holds a promising future in clinical dentistry.

## Data availability statement

The raw data supporting the conclusions of this article will be made available by the authors, without undue reservation.

## Author contributions

JA contributed to the conceptualization, data curation, formal analysis, investigation, methodology, validation, visualization and writing - original draft. YS contributed to the investigation, methodology, software and validation. JZ contributed to the data curation, project administration, resources, validation and visualization. BX contributed to the conceptualization, data curation, project administration, resources, supervision, validation, visualization and writing - review & editing. All authors contributed to the article and approved the submitted version.

## References

[B1] Aati S. Shrestha B. Fawzy A. (2022). Cytotoxicity and antimicrobial efficiency of ZrO2 nanoparticles reinforced 3D printed resins. Dent. Mater. 38 (8), 1432–1442. doi: 10.1016/j.dental.2022.06.030 35792014

[B2] Adnan M. Rahman T. U. Bahadur A. Zeb M. A. Liaqat W. Akitsu T.. (2021). The effect of AlI_3_ nanoadditive on the thermal behavior of PMMA subjected to thermoanalytical py-GC-MS technique. Materials (Basel). 14 (22), 7036. doi: 10.3390/ma14227036 34832436 PMC8624407

[B3] Akhtar A. N. Murtaza G. Shafique M. A. Haidyrah A. S. (2021). Effect of Cu ions implantation on structural, electronic, optical and dielectric properties of polymethyl methacrylate (PMMA). Polymers (Basel). 13 (6), 973. doi: 10.3390/polym13060973 33810029 PMC8005113

[B4] Al-Harbi F. A. Abdel-Halim M. S. Gad M. M. Fouda S. M. Baba N. Z. AlRumaih H. S.. (2019). Effect of nanodiamond addition on flexural strength, impact strength, and surface roughness of PMMA denture base. J. Prosthodont. 28 (1), e417–e425. doi: 10.1111/jopr.12969 30353608

[B5] Ambrožič G. Škapin S. D. Žigon M. Crnjak Orel Z. (2011). Poly (zinc dimethacrylate) as a precursor in the low-temperature formation of ZnO nanoparticles. J. Colloid Interface Sci. 360 (2), 370–376. doi: 10.1016/j.jcis.2011.05.025 21640357

[B6] An J. Ding N. Zhang Z. (2022). Mechanical and antibacterial properties of polymethyl methacrylate modified with zinc dimethacrylate. J. Prosthet Dent. 128 (1), 100.e1–100.e8. doi: 10.1016/j.prosdent.2022.04.029 35680479

[B7] An Y. H. Friedman R. J. (1998). Concise review of mechanisms of bacterial adhesion to biomaterial surfaces. J. Biomed. Mater. Res. 43 (3), 338–348. doi: 10.1002/(sici)1097-4636(199823)43:3<338::aid-jbm16>3.0.co;2-b 9730073

[B8] Arun D. Adikari Mudiyanselage D. Gulam Mohamed R. Liddell M. Monsur Hassan N. M. Sharma D. (2020). Does the addition of zinc oxide nanoparticles improve the antibacterial properties of direct dental composite resins? a systematic review. Materials (Basel). 14 (1), 40. doi: 10.3390/ma14010040 33374229 PMC7795203

[B9] Ausiello P. Cassese A. Miele C. Beguinot F. Garcia-Godoy F. Di Jeso B.. (2013). Cytotoxicity of dental resin composites: An *in vitro* evaluation. J. Appl. Toxicol. 33 (6), 451–457. doi: 10.1002/jat.1765 22120598

[B10] Beyth N. Houri-Haddad Y. Baraness-Hadar L. Yudovin-Farber I. Domb A. J. Weiss E. I. (2008). Surface antimicrobial activity and biocompatibility of incorporated polyethylenimine nanoparticles. Biomaterials. 29 (31), 4157–4163. doi: 10.1016/j.biomaterials.2008.07.003 18678404

[B11] Caldas I. P. Alves G. G. Barbosa I. B. Scelza P. de Noronha F. Scelza M. Z. (2019). *In vitro* cytotoxicity of dental adhesives: A systematic review. Dent. Mater. 35 (2), 195–205. doi: 10.1016/j.dental.2018.11.028 30527507

[B12] Cao L. Xie X. Wang B. Weir M. D. Oates T. W. Xu H. H. K.. (2018). Protein-repellent and antibacterial effects of a novel polymethyl methacrylate resin. J. Dent. 79, 39–45. doi: 10.1016/j.jdent.2018.09.007 30248381

[B13] Chen Y. Xu C. Cao L. Wang Y. Fang L. (2013a). Morphology study of peroxide-induced dynamically vulcanized polypropylene/ethylene-propylene-diene monomer/zinc dimethacrylate blends during tensile deformation. J. Phys. Chem. B. 117 (25), 7819–7825. doi: 10.1021/jp403293b 23742700

[B14] Chen Y. Xu C. Liang X. Cao L. (2013b). *In situ* reactive compatibilization of polypropylene/ethylene-propylene-diene monomer thermoplastic vulcanizate by zinc dimethacrylate *via* peroxide-induced dynamic vulcanization. J. Phys. Chem. B. 117 (36), 10619–10628. doi: 10.1021/jp404427w 23981036

[B15] Chrysafi I. Kontonasaki E. Anastasiou A. D. Patsiaoura D. Papadopoulou L. Vourlias G.. (2020). Mechanical and thermal properties of PMMA resin composites for interim fixed prostheses reinforced with calcium *β*-pyrophosphate. J. Mech. Behav. BioMed. Mater. 112, 104094. doi: 10.1016/j.jmbbm.2020.104094 32979608

[B16] Cocco A. R. Cuevas-Suárez C. E. Liu Y. Lund R. G. Piva E. Hwang G. (2020). Anti-biofilm activity of a novel pit and fissure self-adhesive sealant modified with metallic monomers. Biofouling. 36 (3), 245–255. doi: 10.1080/08927014.2020.1748603 32326753 PMC7270982

[B17] da Silva Barboza A. Fang L. K. Ribeiro J. S. Cuevas-Suárez C. E. Moraes R. R. Lund R. G. (2021). Physicomechanical, optical, and antifungal properties of polymethyl methacrylate modified with metal methacrylate monomers. J. Prosthet Dent. 125 (4), 706.e1–706.e6. doi: 10.1016/j.prosdent.2020.12.039 33581867

[B18] Elmergawy F. H. Nassif M. S. El-Borady O. M. Mabrouk M. El-Korashy D. I. (2021). Physical and mechanical evaluation of dental resin composite after modification with two different types of montmorillonite nanoclay. J. Dent. 112, 103731. doi: 10.1016/j.jdent.2021.103731 34192560

[B19] Elzahar H. B. El-Okaily M. S. Khedr M. H. Amgad Kaddah M. El-Shahawy A. A. G. (2022). Novel cold cure acrylic denture base with recycled zirconia nano-fillers that were functionalized by HEMA agent incorporation: Using the sprinkle approach. Int. J. Nanomedicine. 17, 4639–4658. doi: 10.2147/IJN.S374258 36199477 PMC9528916

[B20] Gad M. M. Al-Thobity A. M. Fouda S. M. Näpänkangas R. Raustia A. (2020). Flexural and surface properties of PMMA denture base material modified with thymoquinone as an antifungal agent. J. Prosthodont. 29 (3), 243–250. doi: 10.1111/jopr.12967 30178899

[B21] Gad M. M. Fouda S. M. Al-Harbi F. A. Näpänkangas R. Raustia A. (2017). PMMA denture base material enhancement: A review of fiber, filler, and nanofiller addition. Int. J. Nanomedicine. 12, 3801–3812. doi: 10.2147/IJN.S130722 28553115 PMC5440038

[B22] Hamid S. K. Alghamdi L. A. Alshahrani F. A. Khan S. Q. Matin A. Gad M. M. (2021). *In vitro* assessment of artificial aging on the antifungal activity of PMMA denture base material modified with ZrO_2_ nanoparticles. Int. J. Dent. 2021, 5560443. doi: 10.1155/2021/5560443 34093706 PMC8137292

[B23] Henn S. de Carvalho R. V. Ogliari F. A. de Souza A. P. Line S. R. da Silva A. F.. (2012). Addition of zinc methacrylate in dental polymers: MMP-2 inhibition and ultimate tensile strength evaluation. Clin. Oral. Investig. 16 (2), 531–536. doi: 10.1007/s00784-011-0551-x 21448634

[B24] Henn S. Nedel F. de Carvalho R. V. Lund R. G. Cenci M. S. Pereira-Cenci T.. (2011). Characterization of an antimicrobial dental resin adhesive containing zinc methacrylate. J. Mater Sci. Mater Med. 22 (8), 1797–1802. doi: 10.1007/s10856-011-4364-x 21670999

[B25] Issa Y. Watts D. C. Brunton P. A. Waters C. M. Duxbury A. J. (2004). Resin composite monomers alter MTT and LDH activity of human gingival fibroblasts *in vitro* . Dent. Mater. 20 (1), 12–20. doi: 10.1016/s0109-5641(03)00053-8 14698769

[B26] Kallem P. Bharath G. Rambabu K. Srinivasakannan C. Banat F. (2021). Improved permeability and antifouling performance of polyethersulfone ultrafiltration membranes tailored by hydroxyapatite/boron nitride nanocomposites. Chemosphere. 268, 129306. doi: 10.1016/j.chemosphere.2020.129306 33360002

[B27] Kanie T. Arikawa H. Fujii K. Inoue K. (2004). Physical and mechanical properties of PMMA resins containing gamma-methacryloxypropyltrimethoxysilane. J. Oral. Rehabil. 31 (2), 166–171. doi: 10.1111/j.1365-2842.2004.01043.x 15009602

[B28] Karatepe U. Y. Ozdemir T. (2020). Improving mechanical and antibacterial properties of PMMA *via* polyblend electrospinning with silk fibroin and polyethyleneimine towards dental applications. Bioact. Mater. 5 (3), 510–515. doi: 10.1016/j.bioactmat.2020.04.005 32322761 PMC7163214

[B29] Kostić M. Igić M. Gligorijević N. Nikolić V. Stošić N. Nikolić L. (2022). The use of acrylate polymers in dentistry. Polymers. 14 (21), 4511. doi: 10.3390/polym14214511 36365504 PMC9653800

[B30] Krasowska A. Sigler K. (2014). How microorganisms use hydrophobicity and what does this mean for human needs? Front. Cell. Infect. Microbiol. 4. doi: 10.3389/fcimb.2014.00112 PMC4137226 25191645

[B31] Lapinska B. Szram A. Zarzycka B. Grzegorczyk J. Hardan L. Sokolowski J.. (2020). An *In vitro* study on the antimicrobial properties of essential oil modified resin composite against oral pathogens. Materials (Basel). 13 (19), 4383. doi: 10.3390/ma13194383 33019681 PMC7579242

[B32] Lee J. H. Jo J. K. Kim D. A. Patel K. D. Kim H. W. Lee H. H. (2018). Nano-graphene oxide incorporated into PMMA resin to prevent microbial adhesion. Dent. Mater. 34 (4), e63–e72. doi: 10.1016/j.dental.2018.01.019 29402540

[B33] Matsuo H. Suenaga H. Takahashi M. Suzuki O. Sasaki K. Takahashi N. (2015). Deterioration of polymethyl methacrylate dentures in the oral cavity. Dent. Mater J. 34 (2), 234–239. doi: 10.4012/dmj.2014-089 25740307

[B34] Melo M. A. Wu J. Weir M. D. Xu H. H. (2014). Novel antibacterial orthodontic cement containing quaternary ammonium monomer dimethylaminododecyl methacrylate. J. Dent. 42 (9), 1193–1201. doi: 10.1016/j.jdent.2014.07.006 25035230 PMC4559222

[B35] Muhammad N. Sarfraz Z. Zafar M. S. Liaqat S. Rahim A. Ahmad P.. (2022). Characterization of various acrylate based artificial teeth for denture fabrication. J. Mater. Sci. Mater. Med. 33 (2), 17. doi: 10.1007/s10856-022-06645-8 35072817 PMC8786782

[B36] Nonkumwong J. Erasquin U. J. Sy Piecco K. W. Premadasa U. I. Aboelenen A. M. Tangonan A.. (2018). Successive surface reactions on hydrophilic silica for modified magnetic nanoparticle attachment probed by sum-frequency generation spectroscopy. Langmuir. 34 (43), 12680–12693. doi: 10.1021/acs.langmuir.8b01333 30300547

[B37] Pei Y. Liu H. Yang Y. Yang Y. Jiao Y. Tay F. R.. (2018). Biological activities and potential oral applications of n-acetylcysteine: Progress and prospects. Oxid. Med. Cell Longev. 2018, 2835787. doi: 10.1155/2018/2835787 29849877 PMC5937417

[B38] Pereira-Cenci T. Deng D. M. Kraneveld E. A. Manders E. M. Del Bel Cury A. A. Ten Cate J. M.. (2008). The effect of streptococcus mutans and candida glabrata on candida albicans biofilms formed on different surfaces. Arch. Oral. Biol. 53 (8), 755–764. doi: 10.1016/j.archoralbio.2008.02.015 18395698

[B39] Pourhajibagher M. Rahimi Esboei B. Hodjat M. Bahador A. (2020). Sonodynamic excitation of nanomicelle curcumin for eradication of streptococcus mutans under sonodynamic antimicrobial chemotherapy: Enhanced anti-caries activity of nanomicelle curcumin. Photodiagnosis Photodyn. Ther. 30, 101780. doi: 10.1016/j.pdpdt.2020.101780 32315777

[B40] Rubin Cocco A. de Oliveira da Rosa W. L. Luque Peralta S. Timm Maske T. da Silva A. F. Andrade Hartwig C.. (2018). New adhesive system based in metals cross-linking methacrylate. J. Mech. Behav. BioMed. Mater. 77, 519–526. doi: 10.1016/j.jmbbm.2017.10.010 29040963

[B41] Vedhanayagam M. Anandasadagopan S. Nair B. U. Sreeram K. J. (2020). Polymethyl methacrylate (PMMA) grafted collagen scaffold reinforced by PdO-TiO_2_ nanocomposites. Mater. Sci. Eng. C Mater. Biol. Appl. 108, 110378. doi: 10.1016/j.msec.2019.110378 31924005

[B42] Walczak K. Schierz G. Basche S. Petto C. Boening K. Wieckiewicz M. (2020). Antifungal and surface properties of chitosan-salts modified PMMA denture base material. Molecules. 25 (24), 5899. doi: 10.3390/molecules25245899 33322112 PMC7763281

[B43] Walters N. J. Xia W. Salih V. Ashley P. F. Young A. M. (2016). Poly (propylene glycol) and urethane dimethacrylates improve conversion of dental composites and reveal complexity of cytocompatibility testing. Dent. Mater. 32 (2), 264–277. doi: 10.1016/j.dental.2015.11.017 26764174

[B44] Wu X. Z. Cheng A. X. Sun L. M. Sun S. J. Lou H. X. (2009). Plagiochin e, an antifungal bis(bibenzyl), exerts its antifungal activity through mitochondrial dysfunction-induced reactive oxygen species accumulation in candida albicans. Biochim. Biophys. Acta 1790 (8), 770–777. doi: 10.1016/j.bbagen.2009.05.002 19446008

[B45] Wu M. Yang L. Zheng Z. Wan F. Teng X. Xu C. (2022). Strengthened self-healable natural rubber composites based on carboxylated cellulose nanofibers participated in ionic supramolecular network. Int. J. Biol. Macromol 222 (Pt A), 587–598. doi: 10.1016/j.ijbiomac.2022.09.192 36167103

[B46] Xia L. Meng J. Ma Y. Zhao P. (2021). Facile fabrication of eucommia rubber composites with high shape memory performance. Polymers (Basel). 13 (20), 3479. doi: 10.3390/polym13203479 34685238 PMC8541577

[B47] Xu C. Cao L. Lin B. Liang X. Chen Y. (2016). Design of self-healing supramolecular rubbers by introducing ionic cross-links into natural rubber *via* a controlled vulcanization. ACS Appl. Mater Interfaces. 8 (27), 17728–17737. doi: 10.1021/acsami.6b05941 27337545

[B48] Yang Y. Zheng M. Jia Y. N. Li J. Li H. P. Tan J. G. (2021). Time-dependent reactive oxygen species inhibit streptococcus mutans growth on zirconia after a helium cold atmospheric plasma treatment. Mater Sci. Eng. C Mater Biol. Appl. 120, 111633. doi: 10.1016/j.msec.2020.111633 33545816

[B49] Yin S. Sun N. Jiang F. Lu Y. Yang G. Wu X.. (2021). The translation from *In vitro* bioactive ion concentration screening to *In vivo* application for preventing peri-implantitis. ACS Appl. Mater Interfaces. 13 (4), 5782–5794. doi: 10.1021/acsami.0c19698 33464812

[B50] Zafar M. S. (2020). Prosthodontic applications of polymethyl methacrylate (PMMA): An update. Polymers. 12 (10), 2299. doi: 10.3390/polym12102299 33049984 PMC7599472

[B51] Zimudzi T. J. Feldman K. E. Sturnfield J. F. Roy A. Hickner M. A. Stafford C. M. (2018). Quantifying carboxylic acid concentration in model polyamide desalination membranes *via* Fourier transform infrared spectroscopy. Macromolecules. 51, 6623–6629. doi: 10.1021/acs.macromol.8b01194 PMC6459611 30983631

[B52] Zou F. Zhou H. Jeong D. Y. Kwon J. Eom S. U. Park T. J.. (2017). Wrinkled surface-mediated antibacterial activity of graphene oxide nanosheets. ACS Appl. Mater Interfaces. 9 (2), 1343–1351. doi: 10.1021/acsami.6b15085 28004574

